# Management of Rhinomaxillary Mucormycosis: A Case Report

**DOI:** 10.7759/cureus.82299

**Published:** 2025-04-15

**Authors:** Sandeep Khandaitkar, Komal Harde, Sharjeel H Khan, Gagandeep Lamba

**Affiliations:** 1 Department of Oral and Maxillofacial Surgery, Ranjeet Deshmukh Dental College and Research Centre, Nagpur, IND; 2 Department of Oral and Maxillofacial Surgery, Swargiya Dadasaheb Kalmegh (SDK) Smruti Dental College and Hospital, Nagpur, IND; 3 Department of Forensic Medicine, Narendra Kumar Prasadrao (NKP) Salve Institute of Medical Sciences and Research Centre, Nagpur, IND; 4 Department of Pediatric and Preventive Dentistry, Ranjeet Deshmukh Dental College and Research Centre, Nagpur, IND

**Keywords:** ampho-b, early debridement, functional endoscopic sinus surgery (fess), rhinomaxillary mucormycosis, total maxillectomy

## Abstract

This case report describes a rare occurrence of palatal mucormycosis in a 45-year-old immunocompetent female patient who developed black discoloration of the palate following a tooth extraction. Initial diagnostic investigations and superficial biopsy revealed aspergillosis; however, further postoperative histopathological examination confirmed mucormycosis. Aggressive surgical intervention, including maxillectomy, alveolectomy, and functional endoscopic sinus surgery, combined with targeted antifungal therapy, facilitated successful treatment. The importance of timely diagnosis and comprehensive management in mucormycosis cases, even among immunocompetent patients, is highlighted.

## Introduction

Mucormycosis, an aggressive fungal infection caused by fungi belonging to the order Mucorales, commonly affects immunocompromised patients [1]. Predominantly seen in individuals with diabetes mellitus, hematologic malignancies, or prolonged immunosuppressive therapy, its occurrence in healthy immunocompetent individuals remains rare [2]. Recent evidence indicates increasing cases of post-dental procedures due to disruption of mucosal integrity, facilitating fungal inoculation [3]. Given its rapid progression and high mortality if untreated, early recognition and aggressive therapeutic strategies are crucial [4]. Initially, owing to its abundant blood supply and close anatomical relationship with the maxillary sinus, the palatal region can be particularly prone to infections, especially after dental extractions or operative procedures, as highlighted by this case report detailing a rare manifestation of palatal mucormycosis in an immunocompetent patient, thereby reinforcing the importance of increased diagnostic awareness in comparable clinical presentations.

## Case presentation

**Figure 1:**
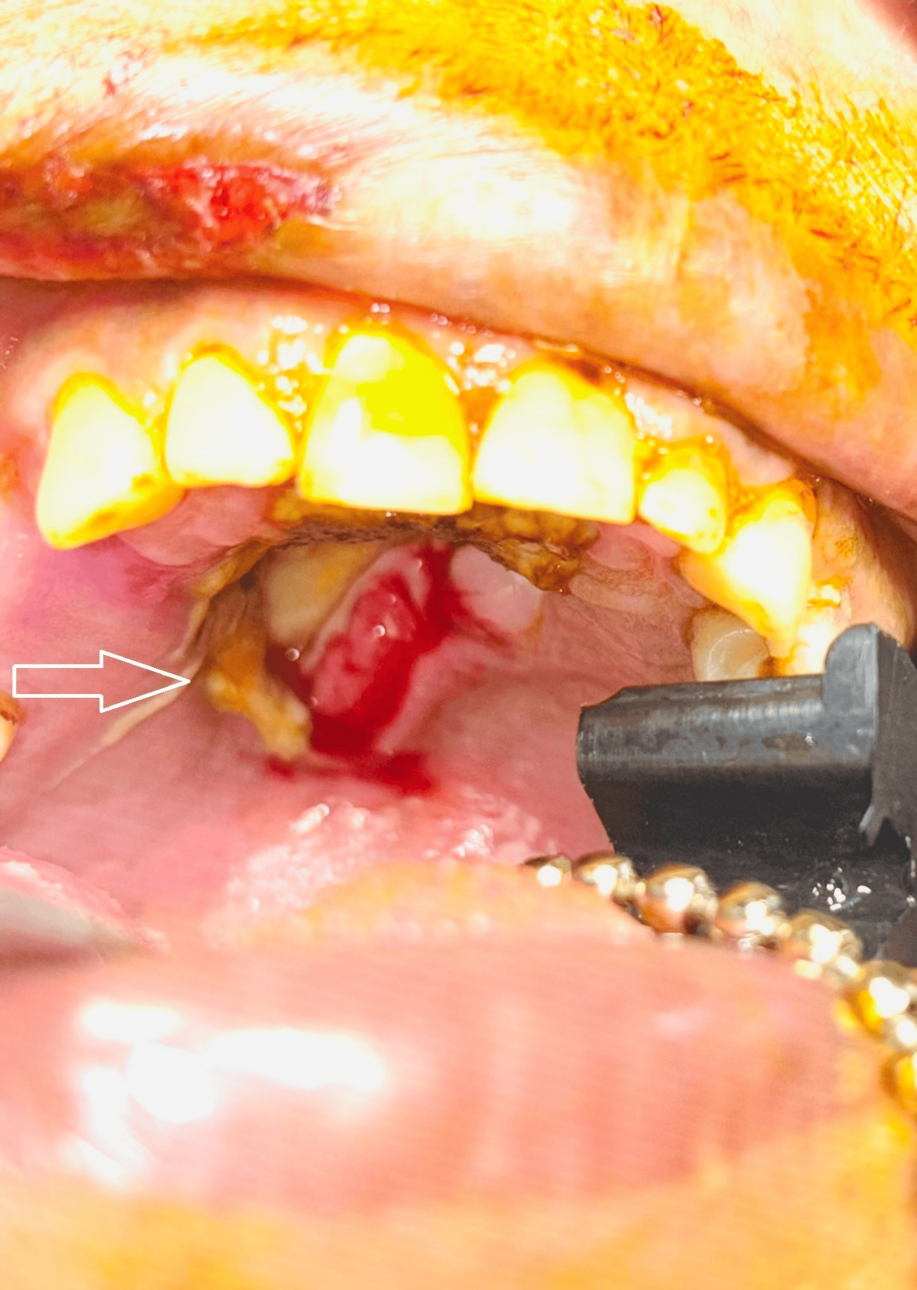
Preoperative intraoral image showing necrotic black eschar (~2 × 2 cm) on the hard palate with surrounding inflammation and exposed bone.

A 45-year-old woman presented to our clinic with a 15-day history of black discoloration on her hard palate following the extraction of an upper right first molar. She reported progressive lesion enlargement, mild pain, and a foul odor emanating from the affected area. Clinical examination revealed a clearly demarcated necrotic black eschar (~2 × 2 cm) on the hard palate with surrounding inflammation and exposed bone, as shown in Figure 1.h

The patient had no notable medical history (no diabetes, human immunodeficiency virus, or immunosuppressive therapy). She also reported no recent illnesses such as COVID-19, chikungunya, or dengue fever. Routine blood investigations, CD4 counts, immunoglobulins, and HbA1c were unremarkable, with no evidence of immunosuppression or hyperglycemia.

**Figure 2:**
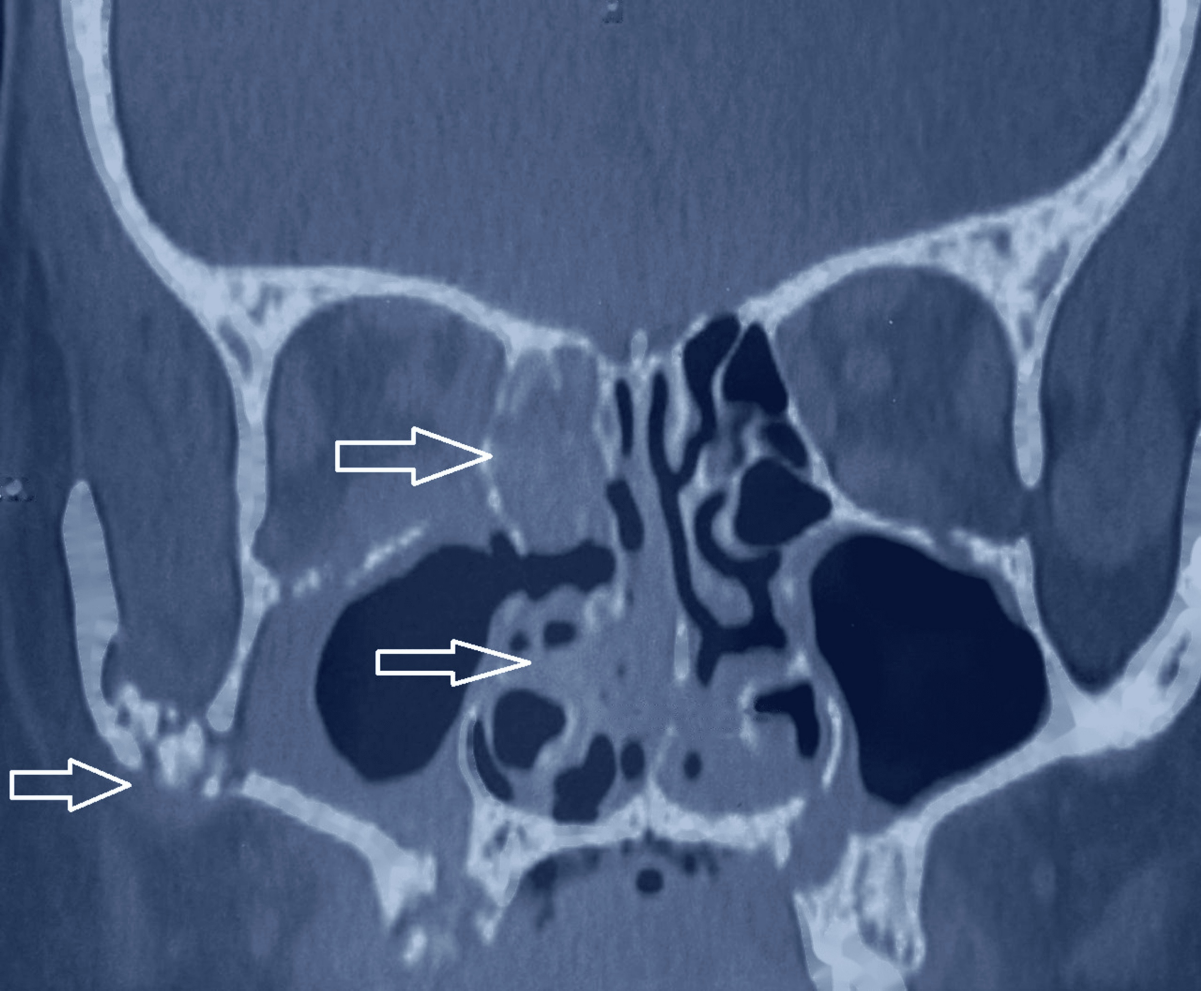
Coronal CT scan (preoperative) displaying the paranasal sinus region, showing opacification of the right maxillary sinus and loss of bony definition in the posterior and medial maxillary wall. These findings indicate sinus opacification with bony erosion and extensive soft tissue density mucosal thickening of the right ethmoidal cells, the frontal sinuses, and the sphenoid sinuses. CT: computed tomography

**Figure 3:**
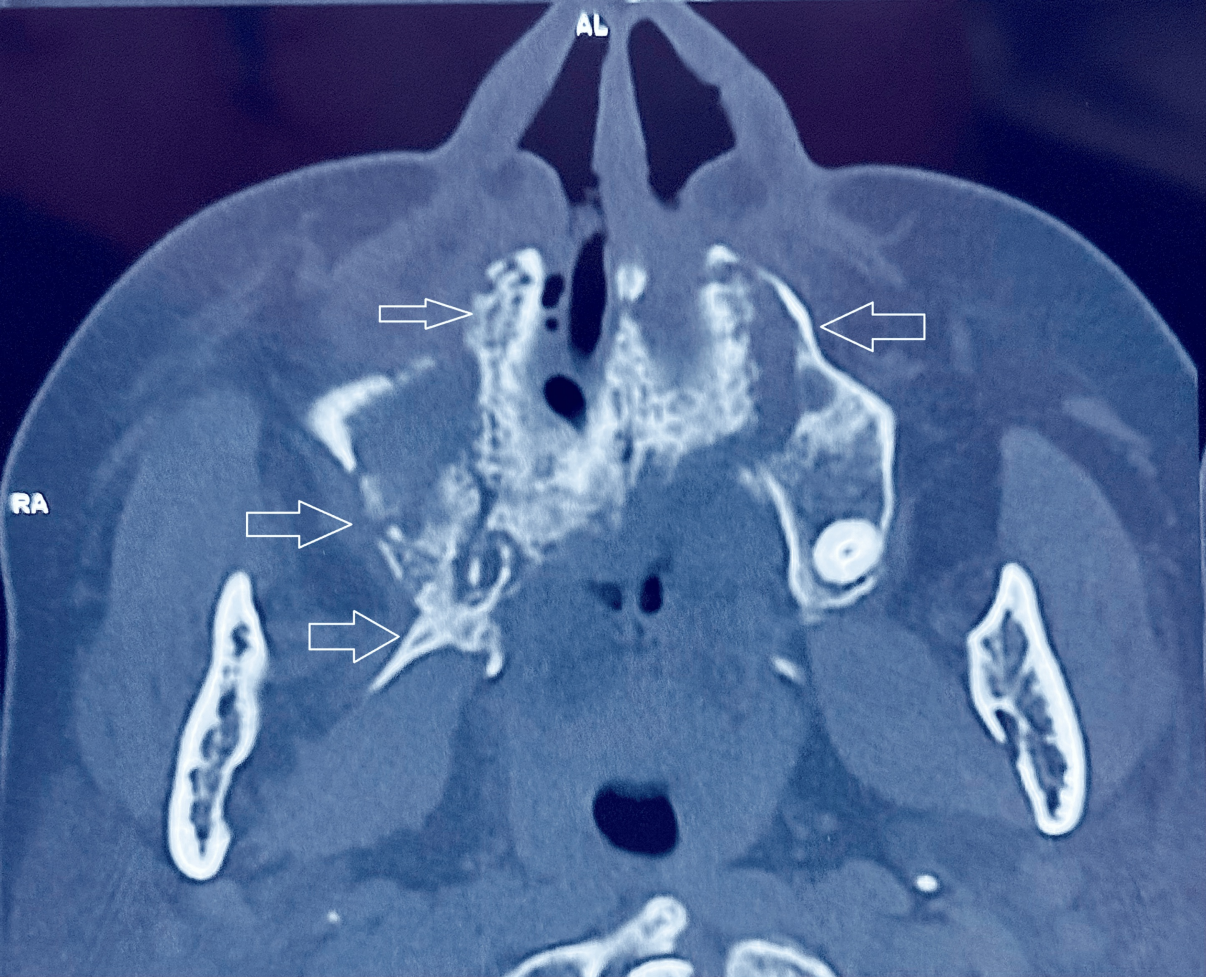
Axial CT section revealing bony erosion of the hard palate, right maxillary alveolus, and right pterygoid plate, as well as the left maxillary alveolar process. CT: computed tomography

The computed tomography (CT) scan of the paranasal sinuses demonstrated significant erosive changes in the maxillary bone, involving both maxillary sinuses and palatal bone. These radiographic findings are illustrated in Figure 2 and Figure 3.

The first histopathological evaluation of a superficial palatal biopsy suggested aspergillosis due to the presence of septate fungal hyphae. Based on the above clinical, radiological, and primitive superficial biopsy, surgical removal in the form of a right partial maxillectomy and alveolectomy was planned.

**Figure 4:**
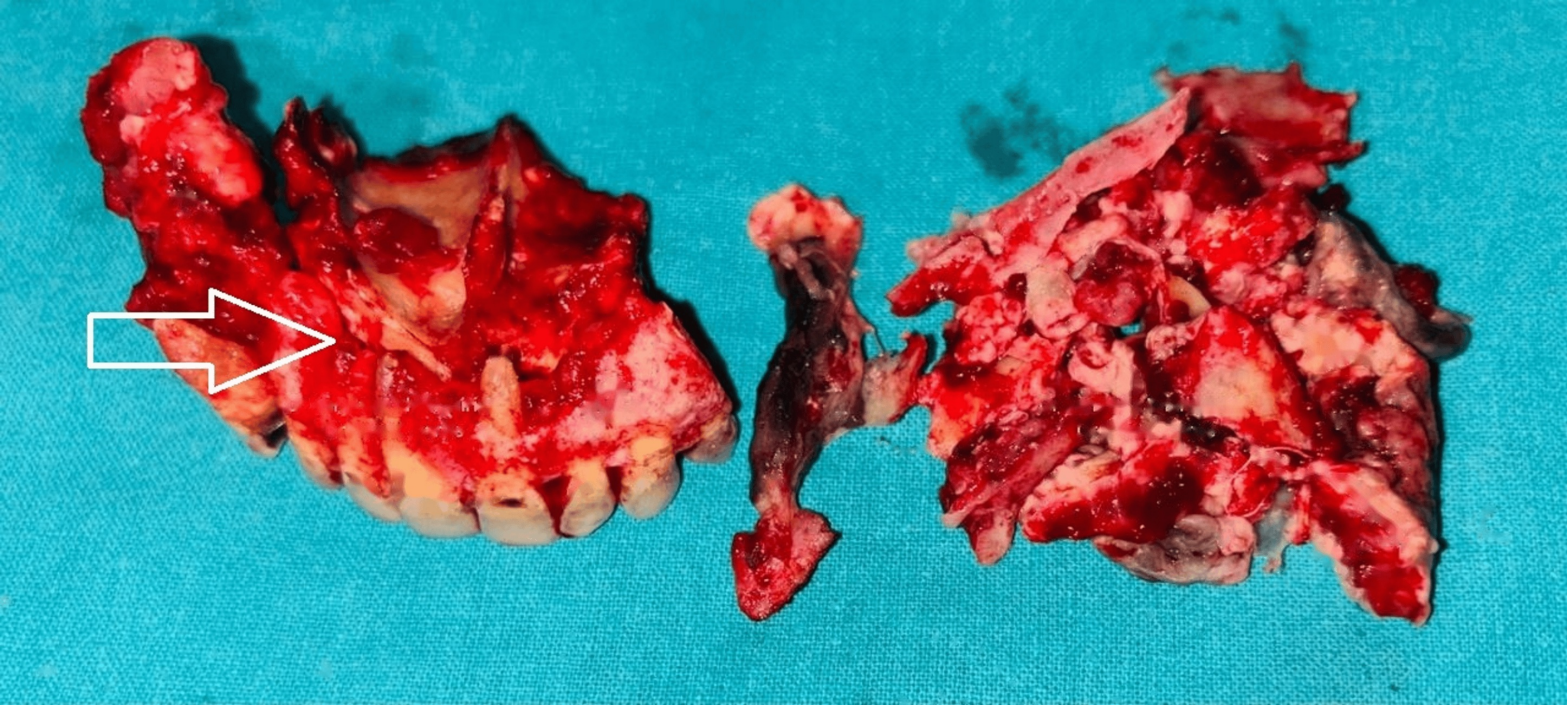
Intraoperative maxillectomy specimen: the resected maxillary segment obtained during surgical debridement is shown, including part of the maxillary alveolus with anterior teeth and necrotic soft tissue. The blackish discoloration of the excised tissue is characteristic of devitalized tissue in mucormycosis, indicating extensive fungal invasion in the specimen.

**Figure 5:**
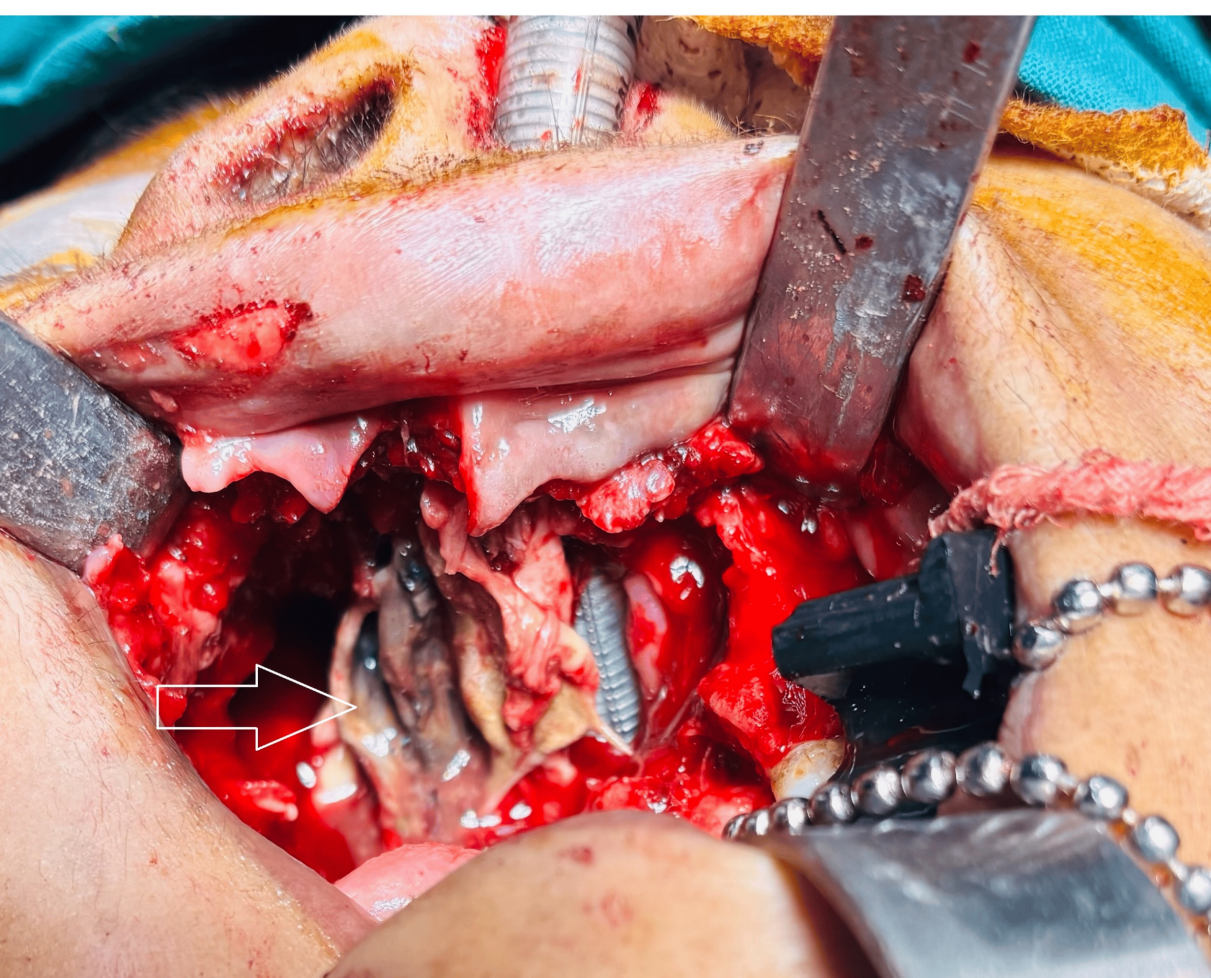
Intraoperative intraoral image showing necrosed inferior turbinates.

The patient underwent extensive surgical debridement, including a right partial maxillectomy and left alveolectomy, alongside functional endoscopic sinus surgery (FESS). Specifically, the resection extended to involve the nasal floor, evident on the axial and coronal CT images (as indicated by arrows), demonstrating destruction and loss of continuity of bone between the maxillary sinus and nasal cavity. Intraoperative examination confirmed complete excision of all necrotic tissue, and the resected maxillary segment is shown in Figure 4 and Figure 5.

**Figure 6:**
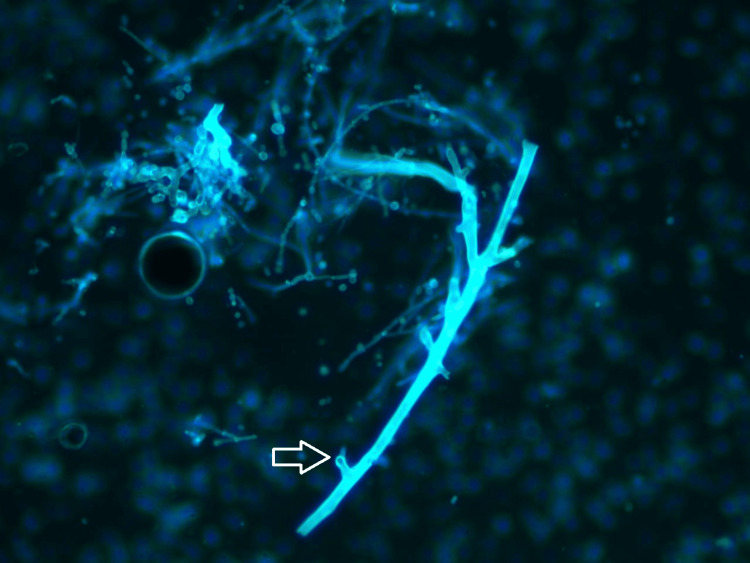
Fluorescent microscopic view of fungal hyphae (Blankophor stain): a fluorescent microscopy image of an intraoperative tissue sample treated with KOH and Blankophor stain, highlighting fungal hyphae. The hyphae appear as bright blue-white, broad ribbon-like structures with right-angle branching, consistent with Mucorales fungi. Scattered debris and spores are also visible. This staining technique rapidly identifies fungal elements by binding to chitin in the fungal cell walls, aiding prompt diagnosis of invasive mucormycosis.

The primary closure of the surgical defect was achieved. A fluorescent potassium hydroxide (KOH) preparation with Blankophor stain of the intraoperative tissue highlighted the characteristic fungal hyphae way evident in Figure 6.

A postoperative histopathology of the intraoperatively resected bony segment confirmed the diagnosis of mucormycosis.

Postoperatively, the patient received intravenous liposomal amphotericin B (5 mg/kg/day) for two weeks, followed by a transition to oral posaconazole (300 mg daily). Adjunctive broad-spectrum antibiotics were administered to prevent secondary bacterial infections.

The postoperative recovery was uneventful, with substantial clinical improvement noted by the third postoperative day. Follow-up examinations showed excellent wound healing without any evidence of recurrence. At three and six months post-surgery, clinical and radiological assessments confirmed sustained remission. Prosthetic rehabilitation was planned to further improve the patient's functional outcome.

## Discussion

Mucormycosis is an invasive fungal infection predominantly associated with immunocompromised patients; however, rare occurrences in immunocompetent individuals present significant diagnostic and therapeutic challenges [5]. The current case highlights mucormycosis in an immunocompetent patient following a routine dental extraction, underscoring that breaches in mucosal integrity can provide an opportunistic portal of entry for pathogenic fungi such as Mucorales [3].

The initial histopathological diagnosis was suggestive of aspergillosis due to the identification of septate hyphae in superficial biopsy samples. Mucormycosis typically infiltrates deeper tissues and thus requires deeper and extensive tissue sampling to reliably detect the broad, non-septate hyphae characteristic of Mucorales species. However, clinical progression and subsequent deep tissue examination confirmed mucormycosis characterized by broad, non-septate hyphae with typical right-angle branching [6]. Additionally, the application of specialized fungal stains, such as Blankophor or periodic acid-Schiff (PAS)/Gomori methenamine silver (GMS), and fungal cultures are crucial diagnostic tools, as they clearly differentiate non-septate hyphae (indicative of Mucorales) from septate hyphae (typical of *Aspergillus* species). Although fungal culture was not conducted in this case, the subsequent deeper tissue sampling combined with histopathological examination using appropriate fungal stains conclusively identified non-septate hyphae consistent with mucormycosis. Such diagnostic ambiguity necessitates vigilance and underscores the importance of multiple, deeper biopsy samples coupled with special fungal staining techniques, as superficial samples might be misleading due to colonization by non-invasive fungi or mixed infections [7].

The aggressive nature of mucormycosis mandates prompt surgical intervention, which has been shown to significantly reduce morbidity and mortality. In the presented case, extensive surgical debridement involving partial maxillectomy, alveolectomy, and functional endoscopic sinus surgery successfully eradicated the infection, thus aligning with established recommendations advocating for aggressive surgical management [8].

Amphotericin B, particularly in its liposomal formulation, remains the cornerstone antifungal agent for mucormycosis. It demonstrates rapid fungicidal activity, favorable tissue penetration, and reduced nephrotoxicity compared to conventional forms [9]. Additionally, the subsequent transition to posaconazole, a broad-spectrum triazole, facilitated prolonged antifungal coverage, thereby reducing the risk of recurrence [10].

Recent literature emphasizes a multidisciplinary approach involving maxillofacial surgeons, otolaryngologists, infectious disease specialists, pathologists, and prosthodontists as essential for optimal outcomes, particularly in managing complex anatomical and functional deficits post-surgery [11]. Prosthetic rehabilitation further enhances patient quality of life by restoring function and aesthetics, critical components frequently compromised in extensive mucormycosis surgeries [12].

The rarity of mucormycosis among immunocompetent individuals demands increased clinical awareness, particularly in post-procedural scenarios where mucosal barriers are disrupted. Early suspicion, accurate diagnosis, aggressive surgical debridement, appropriate antifungal therapy, and coordinated multidisciplinary care are pivotal for favorable patient outcomes.

## Conclusions

In conclusion, this case underscores the critical importance of maintaining a high index of suspicion for mucormycosis, even in immunocompetent patients who present with atypical postoperative palatal lesions following dental procedures. The initial misdiagnosis of aspergillosis highlights the limitations of superficial biopsy and emphasizes the necessity for deeper, extensive tissue sampling accompanied by specialized fungal staining techniques, such as Blankophor, periodic acid-Schiff (PAS), or Gomori methenamine silver (GMS), to reliably distinguish between septate (*Aspergillus*) and non-septate (Mucorales) hyphae. The exact extent of resection, clearly delineated through advanced imaging, involved partial maxillectomy and alveolectomy with significant involvement of the nasal floor, although orbital floor involvement was absent. Prompt diagnosis, aggressive surgical intervention, systemic antifungal therapy, and coordinated multidisciplinary management involving maxillofacial surgery, infectious disease specialists, radiologists, and pathologists significantly enhance patient outcomes by limiting disease progression, reducing morbidity, and improving survival and quality of life. Therefore, clinicians must remain vigilant for such atypical presentations and adopt an integrated diagnostic and therapeutic approach for the effective management of mucormycosis.
